# Promoting Sexual Health in Colorectal Cancer Patients and Survivors: Results from a Systematic Review

**DOI:** 10.3390/healthcare12020253

**Published:** 2024-01-19

**Authors:** Hélia B. Rocha, Bruna C. Carneiro, Priscila A. Vasconcelos, Raquel Pereira, Ana Luísa Quinta-Gomes, Pedro J. Nobre

**Affiliations:** Centre for Psychology at the University of Porto, Faculty of Psychology and Education Sciences, University of Porto, 4200-135 Porto, Portugal; up201604151@up.pt (H.B.R.); up201508751@edu.fpce.up.pt (B.C.C.); up201203768@up.pt (P.A.V.); arlpereira@fpce.up.pt (R.P.); pnobre@fpce.up.pt (P.J.N.)

**Keywords:** colorectal cancer, oncosexology, systematic review, sexual health, e-health, eMental health, psychological interventions

## Abstract

Background: Colorectal cancer diagnosis and treatment negatively impact sexual health. However, there is still a lack of interventions targeting the sexual healthcare needs of colorectal cancer patients and survivors. This systematic review aimed to identify and summarize the efficacy of available psychological interventions aimed at improving colorectal cancer patients’ and survivors’ sexual health. Methods: This review followed PRISMA guidelines for systematic reviews. A database search was conducted for studies published until July 2023 on EBSCO Host, Web of Science, PubMed, and the Cochrane Library. Manuscripts were screened according to inclusion and exclusion criteria. The risk of bias was assessed using the Quality Assessment Tool for Quantitative Studies. Results: From the 1499 records screened, four studies describing psychological interventions to improve the sexual health of the target population were identified. All studies reported on e-health programs and showed evidence of their efficacy in the improvement of participants’ sexual function. The studies presented low scores on the Quality Assessment Tool for Quantitative Studies. Conclusions: Despite the evidence that tele/e-health psychological interventions have the potential to effectively promote sexual health in colorectal cancer patients and survivors, more robust research is needed to allow for generalization. Future research should further assess the efficacy of e-health interventions (eMental Health) in promoting sexual health in patients with colorectal cancer.

## 1. Introduction

Colorectal cancer (CRC) is the fourth most commonly diagnosed type of cancer worldwide and the second with the highest mortality rate [1]. Early screening and treatment advances have contributed to reduced CRC-related mortality in countries with a higher human development index [2]. Despite the growing survival rates and the importance of preserving survivors’ quality of life and well-being, guidelines regarding the management of long-term symptoms and sequelae of treatments are still lacking [3].

Conventional treatments for CRC include surgery, chemotherapy, and radiation therapy [4], which can lead to the onset of a variety of physical, emotional, psychological, and social impairments [5,6,7,8], impacting patients’ overall quality of life and sexual well-being throughout the course of the disease [8,9]. In the case of stoma surgery, such an impact may be more dramatic and exacerbate overall symptoms, posing additional challenges in the patient’s adjustment to the new condition [10].

Regarding the impact on sexual health, several studies have shown CRC and associated treatments can precipitate the development of sexual problems, such as erectile and ejaculatory dysfunction in men and dyspareunia and absent or decreased vaginal lubrication in women [11,12,13], along with a significant increase in sexual distress in both patients and partners over time [14]. Sexual desire, sexual pleasure, sexual satisfaction, and the frequency of sexual activities have also been reported to decrease over the course of the disease [10,11,12,15]. In addition to these difficulties, ostomized patients can also experience several constraints with their body image and the interference of the ostomy pouch during sexual activity [16], which can further aggravate their sexual distress and difficulties.

Despite the well-documented impact of CRC diagnosis and treatments on patient’s sexual health [10,11,12,13,17], research seeking to identify the specific sexual healthcare needs of these patients is still lacking [14,18]. A recent study conducted by Carneiro [19] indicated that patients with a history of CRC reported several sexual health concerns, namely regarding physical changes due to cancer and associated treatments, changes in sexual functioning, the ability to have sexual pleasure, and the ability to satisfy the sexual partner. In addition, participants also reported sexual health concerns and needs related to their intimate relationships, such as needs for greater emotional closeness, sexual compatibility and communication with the partner, and concerns about partners’ sexual difficulties. Despite the impact of CRC and its treatments on the quality of the intimate and sexual relationships between patients and their partners [20,21], these sexual healthcare needs [18,22,23] are still not being adequately and routinely addressed in oncological care [18].

Given the extent of sexual health problems experienced after CRC, concerns with sexuality should be considered when addressing CRC patients’ and survivors’ intervention needs. In a focus group conducted by Traa et al. [18], CRC patients and their partners identified the importance of addressing the topics of intimacy, relationship quality, and psychological factors in sexual healthcare programs, above and beyond sexual dysfunctions [18]. Healthcare professionals have also been proposed as the ones to take the lead in addressing the issue of sexual health [16]. Healthcare providers may also provide information on the medical and psychological interventions that are available during the course of the disease [24,25]. However, studies have indicated that such information is not being properly communicated to patients [15,16,20]. Flynn et al. [16] reported that 55% of participants with colorectal cancer did not receive information about how cancer and its treatments could affect their sex life. Similarly, Sutsunbuloglu and Vural [15] reported that, of a total of 100 stoma patients (84% had colorectal cancer), 79% had not received any information about the potential side effects of cancer treatment on sexual health, and 83% had not been informed about treatment opportunities for sexual problems.

Given the importance of psychological sexual healthcare provision for CRC patients and survivors, this systematic review aims to identify existing psychological interventions designed to improve the sexual health (i.e., sexual function, sexual well-being, sexual satisfaction, and intimacy) of this population. Although previous systematic reviews have been conducted on psychological interventions to promote sexual health in overall cancer patients [26,27], studies are yet to specifically focus on the sexual health of men and women with CRC. This present review aims to further contribute to the systematization of the literature on this field by identifying interventions specifically designed to improve the sexual health of male and female CRC patients and survivors. To the best of the authors’ knowledge, this is the first systematic review attempting to summarize psychological sexual health interventions designed for CRC patients and survivors.

## 2. Materials and Methods

### 2.1. Protocol and Registration

This systematic review followed the Preferred Reporting Items for Systematic Reviews and Meta-Analyses (PRISMA) guidelines [28]. Pre-registration was performed on Prospero with the number CRD42023442402.

### 2.2. Literature Search Strategy and Study Selection

A protocol was elaborated, resulting in the research question “what are the available psychological interventions aimed at promoting CRC patients and survivors’ sexual health?”. The literature search was conducted on the following databases: EBSCO Host, Web of Science, PubMed, and the Cochrane Library. The databases were searched on July 2023 with no filters applied, namely by publication date, language, or type of publication. The records obtained were exported to Rayyan, and duplicates were removed manually.

The following strings were searched in each database: (1) (online or digital) AND (intervention or program or treatment or therapy) AND (“sexual* health” OR “sexual function” OR “sexual wellbeing” OR “sexual activity” OR “sexual satisfaction” OR intimacy) AND colorectal cancer; (2) (mobile or tele*) AND (intervention or program or treatment or therapy) AND (“sexual* health” OR “sexual function” OR “sexual wellbeing” OR “sexual activity” OR “sexual satisfaction” OR intimacy) AND colorectal cancer; (3) (internet or web) AND (intervention or program or treatment or therapy) AND (“sexual* health” OR “sexual function” OR “sexual wellbeing” OR “sexual activity” OR “sexual satisfaction” OR intimacy) AND colorectal cancer; (4) (intervention or program or treatment or therapy) AND (“sexual* health” OR “sexual function” OR “sexual wellbeing” OR “sexual activity” OR “sexual satisfaction” OR intimacy) AND colorectal cancer.

### 2.3. Eligibility Criteria and Data Extraction

Records were initially screened by title and abstract by one reviewer who identified potential inclusions. Based on the PICO framework, eligibility was decided according to the following criteria: (P) studies where the sample is composed or partially composed of CRC patients or survivors, and, thus, studies not specifying participants’ cancer type were excluded; (I) studies presenting psychological interventions to promote the sexual health of CRC patients and survivors; (C) where applicable, studies including a control sample of adult CRC patients or survivors; and (O) quantitative, qualitative, or mixed-method studies reporting sexual health outcomes (e.g., sexual function, sexual distress) for CRC patients or survivors, thus excluding trials, protocols, and interventions that did not target sexual health outcomes. Additionally, only studies published in English were included. Studies identified as potential inclusions through the first screening were then submitted to a full-text analysis. All authors discussed the eligibility of the potential inclusions and agreed on the final selection of studies for the systematic review.

The risk of bias was assessed using the Quality Assessment Tool for Quantitative Studies [29].

## 3. Results

**Figure 1:**
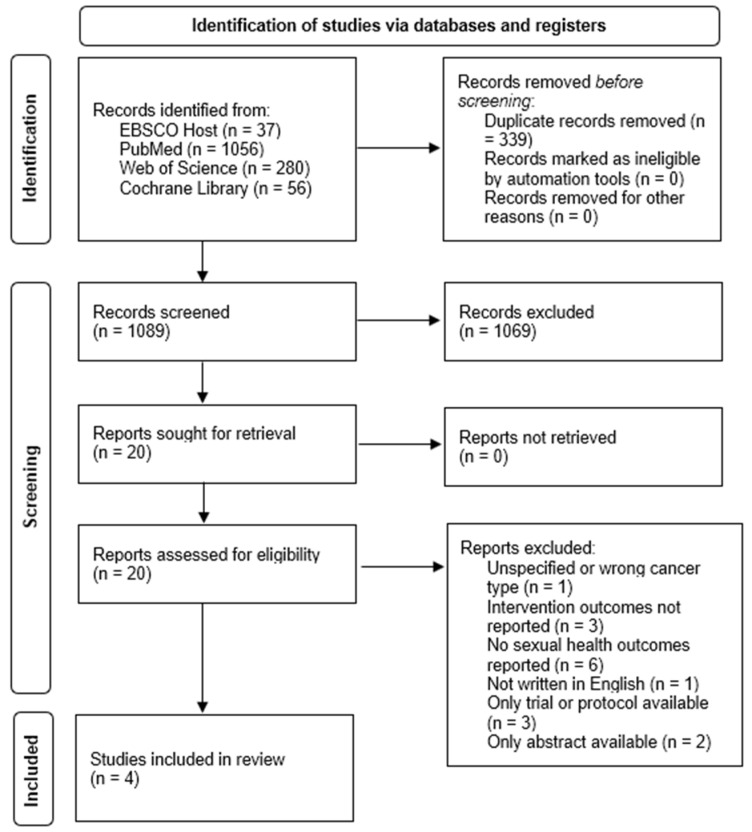
PRISMA Chart.

A total of 1499 records were identified through data search. After the removal of duplicates, 1089 records were screened based on title and abstract, leading to the removal of 1069 records that did not meet the inclusion criteria. The remaining 20 reports were assessed for eligibility through full-text analysis. From this analysis, 16 reports were excluded, resulting in the final 4 studies included in the review (see Figure 1). The studies included are summarized in Table 1.

The following results describe the current available psychological interventions designed to promote CRC patients’ and survivors’ sexual health. To assess the interventions’ outcomes, the following measures were used: Beck Depression Inventory (BDI) [30] by Brotto et al. [31]; Dyadic Adjustment Scale (DAS) [32,33] by Reese et al. [34] and Brotto et al. [31]; Dyadic Sexual Communication Scale (DSCS) [35] by Reese et al. [34,36]; European Organization for Research and Treatment of Cancer Core Quality of Life Questionnaire (EORTC-QLQ-C30) [37] by DuHamel et al. [38]; Female Sexual Distress Scale (FSDS) [39] by Brotto et al. [31]; Female Sexual Function Index (FSFI) [40] by Reese et al. [34,36], Brotto et al. [31], and DuHamel et al. [38]; Impact of Events Scale-Revised (IES-R) [41] by DuHamel et al. [38]; Index of Sexual Satisfaction (ISS) [42] by Reese et al. [34,36]; International Index of Erectile Functioning (IIEF) [43] by Reese et al. [36]; Miller Social Intimacy Scale (MSIS) [44] by Reese et al. [34,36]; Sexual Function Questionnaire (SFQ) [45] by Reese et al. [36]; and the Sexual Beliefs and Information Questionnaire-Revised (SBIQ-R) [46] by Brotto et al. [31].

**Table 1:** Summary of included studies.

Authors (Year), Country	Aims	Study Design	Participants	Intervention	Duration	Measures
Reese et al. (2012) [34], USA	To collect preliminary data on the feasibility and efficacy of a telephone-based couples intimacy enhancement (IE) intervention protocol.	Pilot feasibility study	*N* = 9 couples (CRC patients and partners) Age: *M* = 61.6 (*SD* = 14.5)	Intimacy Enhancement Intervention	Four weekly 50 min telephone-based sessions	Utilization of skills Program evaluation ISS FSFI DSCS MSIS DAS-4
**Outcomes**	**Program evaluation** Considered “quite a bit” easy to participate in, overall helpful, and important for people with CRC for at least 83% of participants; 78% liked the telephone-based format; 72% reported it was “quite a bit” helpful in enhancing intimacy. **Preliminary effect sizes** CCR Patients: ≥0.80 for sexual distress, female sexual function, and sexual communication; between 0.30 and 0.60 for dyadic adjustment; between 0.20 and 0.30 for intimacy and male sexual function. Spouses: >0.80 for female sexual function; between 0.30 and 0.60 for remaining outcomes. **Key Findings** IE Intervention may be feasible and effective for CCR survivors and their spouses.					
Reese et al. (2014) [36], USA	To assess the feasibility, acceptability, and preliminary efficacy of a telephone-based couples IE intervention protocol.	Randomized pilot trial	*N* = 23 couples (CRC patients and partners; IE group *n* = 10, control group *n* = 8) Age: *M* = 52.6 (*SD* = 10.6)	Intimacy Enhancement Intervention	Four weekly 50 min telephone-based sessions	ISS DSCS MSIS FSFI IIEF Medical impact subscale of SFQ Self-Efficacy
**Outcomes**	**Feasibility and acceptability** **Participation**: 10 couples completed the full program. **Program evaluation and skill utilization**: overall favourable program evaluation; skills utilized by most participants during the past 2 weeks. **Preliminary efficacy** **Effect sizes for CRC patients**: large for female sexual function; medium to large for the reduction of medical impact on sexual function, self-efficacy, and male sexual function; no effect on sexual distress nor intimacy; negative for sexual communication and two self-efficacy items. **Effect sizes for partners**: large for sexual communication, male sexual function, and two self-efficacy items; medium to large for sexual distress; medium for medical impact on sexual function, intimacy, and one self-efficacy item. **Key findings** IE intervention may be feasible for improving CRC patients and partners’ physical and emotional intimacy.					
DuHamel et al. (2016) [38], USA	To evaluate the efficacy of pilot, telephone-based Cancer Survivorship Intervention–Sexual Health (CSI-SH)	Randomized controlled trial	*N* = 70 female rectal or anal cancer survivors (*n* = 33 intervention; *n* = 37 usual care) RC = 69.6%	Cancer Survivorship Intervention–Sexual Health (CSI-SH)	Four individual, 1-h, in-person or telephone sessions.	FSFI IES-R BSI EORTC-QLQ-C30
**Outcomes**	**Sexual Functioning and Psychological Distress** **Follow-up 1**: improvement in all scores compared to baseline. **Follow-up 2**: equivalent results to follow-up 1. **Intervention’s effect sizes** **Follow-up 1**: medium range. **Follow-up 2**: QLQ-C30 emotional functioning and FSFI lubrication presented larger effect sizes at follow-up 2; remaining measures had smaller effect sizes than at follow-up 1. **Linear regression** **Follow-up 1**: significant treatment effects for IES total cancer-specific stress and QLQ-C30 emotional functioning, both improved. **Follow-up 2**: significant treatment effect for QLQ-C30 emotional functioning; higher IES total cancer-specific distress found for married participants. **Key findings** CSI-SH does not lead to significant improvements in sexual functioning. CSI-SH may lead to improvements in quality-of-life dimensions. CSI-SH is more beneficial for sexually active women and rectal and anal cancer survivors.					
Brotto et al. (2017) [31], Canada	To assess the efficacy of an online adaptation of a face-to-face psychoeducational and mindfulness-based programme for sexual difficulties.	Quantitative: baseline, follow-up 1, and follow-up 2. No control group. Qualitative: Interviews.	Total *N* = 61 CCR *N* = 8 women, 15 men (the remaining sample has other types of cancer)	Psychoeducational Intervention for Sexual Health in Cancer Survivors (OPES)	12 weekly topics, 60 min per topic	DAS BDI Qualitative interviews.
**Outcomes**	**Program Acceptability** **Duration**: M = 29.9 weeks (SD = 19.4), range 8–111 weeks **Informativeness**: M = 3.62 (SD = 0.74), i.e., “moderately” and “very much”. **Difficulty understanding information**: M = 1.58 (SD = 0.79), i.e., “not at all” and “a little”. **Sex-related distress** **Women**: more destressed at baseline; significant decrease; further reduction at 6-month follow up. **Men**: no significant decrease. **Sexual Functioning** **Women**: significant improvement in all measured aspects. **Men**: significant improvement in intercourse satisfaction. **Dyadic Adjustment** **Women**: no significant change in consensus, satisfaction, nor cohesion. **Men**: significant decrease in consensus, no significant change in satisfaction nor cohesion. **Mood** **Women**: more depressive symptoms at baseline; significant decrease. **Men**: no significant decrease in depressive symptoms. **Qualitative Interviews** Opportunities for self-reflection, newfound sense of intimacy with partners, enjoyment of the online format, enjoyment of homework, and step-by-step instructions. **Key findings** Feasibility was found for an online mindfulness-based intervention for sexual concerns. Female and male cancer survivors may have different intervention needs.					

**Note.** ISS: Index of Sexual Satisfaction, FSFI: Female Sexual Function Index, DSCS: Dyadic Sexual Communication Scale, MSIS: Miller Social Intimacy Scale, DAS: Dyadic Adjustment Scale, IIEF: International Index of Erectile Functioning, SFQ: Sexual Function Questionnaire, IES-R: Impact of Events Scale-Revised, BSI: Brief Symptom Inventory, EORTC-QLQ-C30: European Organization for Research and Treatment of Cancer Core Quality of Life Questionnaire, BDI: Beck’s Depression Inventory.

### 3.1. Intimacy Enhancement (IE) Intervention 

IE was the object of two of the studies included in this systematic review: a pilot feasibility study [34] and a randomized pilot trial [36].

IE is a tele-health, telephone-based couple’s intervention protocol designed for CRC patients who reported sexual concerns on behalf of themselves and their partners. This intervention used strategies from both couple’s and sexual cognitive-behavioural therapies and consisted of four weekly 50 min telephone call sessions, aiming to educate on “behavioural skills for coping with sexual challenges” specific to CRC [34], as well as to improve physical and emotional intimacy [36]. Each session was associated with a specific theme. Session one focused on how CRC affects sex and intimacy, with couples identifying their challenges and defining goals based on them. Session two addressed communication through skill training, i.e., “effective communication”, “speaker–listener roles”, and “identifying communication challenges” [34]. During session three, cognitive restructuring was introduced, as well as behavioural intimacy-enhancement strategies. In turn, session four served as a conclusion to the intervention, aiming to review previous content and anticipate future challenges, discussing how to approach them. Beyond the sessions, participants had “intimacy-building” homework activities designed to review the proposed strategies.

Preliminary findings [34] showed large effect sizes for male CRC patients’ sexual distress and sexual communication, as well as for female CRC patients’ sexual function. Effect sizes were also large for female spouses’ sexual function. In turn, a medium effect size was found for CRC patients’ dyadic adjustment and for spouses’ outcomes. Intimacy and male sexual function of CRC patients presented small effect sizes.

This intervention was later tested on a new sample, and preliminary efficacy results were reported [36]. Large effect sizes were found for CRC patients’ female sexual function and partners’ sexual communication, male sexual function, and two self-efficacy items. The authors reported medium-to-large effect sizes for CRC patients’ medical impact on sexual function, self-efficacy, and male sexual function, as well as for partners’ sexual distress. Medium effect sizes were found for partners’ medical impact on sexual function, intimacy, and one self-efficacy item. No effect sizes were found for CRC patients’ sexual distress and intimacy. Lastly, CRC patients’ sexual communication and two self-efficacy items presented a negative effect size (see Table 1).

### 3.2. Psychoeducational Intervention for Sexual Health in Cancer Survivors (OPES)

OPES is a e-health, digital, self-administered adaptation of Brotto et al.’s [31] in-person program, aimed at women with gynaecologic cancer. The adapted program consists of a website designed for both male and female patients, specifically focusing on gynaecologic, colon, and rectal cancer survivors reporting sexual distress. In this 12-week, mindfulness-based, psychoeducational intervention, a new topic was released each week as a module, which was expected to be completed in one hour. Additionally, participants were expected to complete homework for each module. They also had the opportunity to anonymously submit questions through a discussion board, which were answered by the research team. Participants could not move on to the next module without completing the previous one and would receive reminders in case they failed to finish the module during the given week. Feasibility analysis found, however, that participants took 29 weeks to go through the full program, on average.

The topics covered by the program were as follows: “(1) the importance of sexuality to quality of life and information on the prevalence of sexual difficulties; (2) consideration of the predisposing, precipitating, perpetuating, and protective factors; (3) sexual beliefs/maladaptive thoughts; (4) mindfulness; (5) genital anatomy and physiology; (6) body image; (7) enhancing relationship satisfaction and communication; (8) mindfulness and awareness of body sensations; (9) thought records; (10) mindfulness of thoughts; (11) using sexual aids (e.g., stimulators, erotica) to enhance arousal; and (12) relapse prevention” [31].

Brotto et al. [31] presented quantitative results of two follow-up moments regarding sexual distress, sexual function, dyadic adjustment, and mood. Women reported higher levels of sex-related distress at baseline than men, presenting a significant post-test decrease and a further reduction at a 6-month follow-up. In turn, men did not present statistically significant results for sex-related distress. Concerning sexual function, women reported significant increases in all dimensions, while men only presented significant improvements in intercourse satisfaction. Dyadic adjustment did not significantly vary for women, while men reported a significant decrease in dyadic consensus. Lastly, women presented more depressive symptoms at baseline than men, resulting in a significant decrease in women’s symptoms at the follow-up assessment and no significant outcomes in men’s symptoms.

### 3.3. Cancer Survivorship Intervention–Sexual Health (CSI-SH)

CSI-SH [38] was aimed at female survivors of rectal or anal cancer who reported low or moderate levels of satisfaction with sexual function. During each of the four sessions, the participant and the psycho-oncology clinician would discuss a specific theme. These sessions could be attended in person or in a tele-health format provided via phone, according to the participant’s requirements. In the first session, a general discussion of sexual health, as well as an assessment of the participant’s sexual health, were carried out. During the second session, strategies to promote sexual health and general well-being were discussed. The third session focused on partner communication strategies. Lastly, during the fourth session, participants were provided with additional relevant information (e.g., possible referrals to other professionals). Both the participant and the psycho-oncology clinician had access to an intervention manual. Additionally, there was homework assigned for each session. The discussions resulting from the sessions were expected to serve as a framework for a tailored treatment plan for the participant. Moreover, between sessions, participants would receive booster calls to aid with strategy implementation and adherence.

Participants were assessed in two follow-ups. The results from the 4-month follow-up indicated an improvement in the scores of all measured dimensions, which remained stable at the 8-month follow-up, particularly for women who reported being sexually active. However, outcomes for sexual function were not statistically significant.

### 3.4. Risk of Bias

All studies presented a risk of bias score of “weak” on the global rating of the Quality Assessment Tool for Quantitative Studies [29]. Scores for each dimension are displayed in Table 2.

## 4. Discussion

The deleterious effects of CRC diagnosis and associated treatments on patients’ and survivors’ sexual health are well established [10,11,12,13,17,19]. However, evidence on interventions designed to address the sexual healthcare needs of these patients is still scarce [18], and available studies on the efficacy of psychological interventions have not been previously systematized. This systematic review focused on the available psychological interventions aimed at promoting CRC patients’ and survivors’ sexual health.

A total of four studies that met the inclusion criteria were identified. The interventions adopted in the selected studies were mediated via tele/e-health or presented the option of being delivered using these formats: a telephone-based intervention, an online intervention, and an intervention offering participants the possibility to be conducted in-person or via telephone. The target group of participants enrolled in the studies varied from study to study. One of the studies was directed at CRC patients and their partners, and the remaining three targeted CRC survivors. The diversity found in the adopted methodology across studies and the target group of participants recruited for the studies further reinforces the need for a continuous focus on the sexual health of the individuals who experience CRC [24]. In addition, all the studies have reported participants’ sexual health concerns and presented positive intervention outcomes regardless of the treatment phase participants were in at the time of the study. These findings also highlight the need for a more comprehensive approach that integrates longitudinal methodologies to monitor CRC patients at different treatment stages of their disease [6,9,24,25]. The application of such a research design would not only shed light on the sexual health needs at different stages of CRC treatment but would also allow for exploration of the differential effect of sexual health interventions.

The interventions implemented in the different studies varied in several therapeutic components and content. However, communication skill training between partners was one of the most widely used therapeutic strategies, regardless of whether or not the intervention was aimed at the partners. There is a vast body of literature demonstrating the impact of cancer on the partner and, consequently, on the couples’ intimate relationships and sexuality [14,20,21]. Under such circumstances, new and complex relationship dynamics can emerge and require effective communication skills between the members of the couple, especially if the couple is facing sexual difficulties. In this scenario, some couples may need to renegotiate their sexual practices or intimacy [47] and redefine sexual expression within the relationship [48]. According to these findings and previous studies highlighting the impact of effective communication between partners on the quality of life and psychological and physical outcomes [49], psychological interventions designed to promote sexual health should include communication skill training to facilitate the refinement of communication between the couple and to promote new forms of intimacy, fostering greater understanding and emotional closeness between partners, as well as better adjustment to the disease. Furthermore, as also demonstrated in the study by Perz and Ussher [49], interventions focused on providing information about changes in sexual health to people with cancer and their partners are more effective on couples’ effective communication and their adjustment to cancer.

Overall, these findings suggest a positive impact of couple-based sexual health interventions on couples’ communication and on minimizing the psychological distress of patients and their partners [50,51,52]. Therefore, future sexual health interventions for CRC patients and survivors should consider incorporating effective communication strategies into their content, along with the possibility of actively involving partners in the treatment plan by adopting a dyadic approach to treatment and research. Promoting an environment of open and effective communication between the couple will play a key role in fostering understanding and facilitating cooperation in exploring new strategies for adjusting to the new sexual reality imposed by cancer.

Another common theme that emerged in this study was the need to address patients’ unmet sexual health needs and to contribute to the provision of information and care that facilitates the adjustment of patients’ and couples’ expectations regarding their sexual functioning during the disease trajectory. This is in line with previous suggestions to address sexual health with individuals with CRC [24] by providing information about the potential changes in sexuality resulting from cancer and treatments [25]. However, although communication about sexuality is crucial, studies suggest that information about the potential side effects of cancer and treatments on sexual health has not been adequately addressed in most cancer care units [16,18,19,22]. Therefore, future interventions aimed at this population should provide information in this regard and facilitate the development of skills that will enable CRC patients to cope more adequately with the different sexual challenges during the course of the disease. Digital interventions can play an important role in achieving this goal, as they provide a convenient, effective, and cost-effective means of delivering mental health care (eMental Health) [53,54], overcoming barriers to accessing specialized sexual health support (e.g., fear of stigmatization, time and cost constraints, lack of specialized healthcare providers). The development of e-health programs for CRC patients could be a promising approach to address the unmet sexual healthcare needs of patients and couples and to provide specialized care in an accessible and private way.

### 4.1. Study Limitations

The studies included in this systematic review scored low on the Quality Assessment Tool for Quantitative Studies [29]. Therefore, the results reported for these interventions should be considered cautiously and not be generalized. The methodological weaknesses associated with the studies, such as the use of non-representative samples, the non-guaranteed blinding process, and the high drop-out rates, may stem from the low scores. In addition, only two studies [36,38] included a control group in their study design, and only the studies focusing on IE [34,36] carried out an effect size analysis, which may also have contributed to these results. Future studies should adopt a randomized controlled trial design, provide detailed information on the randomization and blinding process, and control for potential confounding variables. Regarding the target participants, our results indicated the absence of palliative care patients in the existing literature, which possibly stems from the lack of sexual health assessments carried out at this stage in the course of the disease [55]. Strategies to minimize drop-out rates should also be considered, as most studies have indicated high attrition rates.

### 4.2. Clinical Implications

The results of this study highlighted the need to address sexual health continuously and along the cancer trajectory of patients with CRC. Regardless of the delivery mode of the provision of sexual healthcare, (i.e., in-person, telephone-based, digital, or mixed), the findings showed that both patients and partners can benefit from sexual healthcare intervention addressing their specific preferences and needs. Developing e-health programs (eMH) for CRC patients is a promising approach to address the unmet sexual healthcare needs of patients and couples and to provide specialized care in an accessible and private way.

## 5. Conclusions

There is evidence that psychological interventions, particularly tele/e-health interventions, have the potential to promote sexual health in CRC patients and survivors effectively. However, the results of the analysed studies need to be more robust to be generalized, and additional research is needed, including studies with larger and more diverse populations, to obtain more solid results on the efficacy of psychological interventions in this population.

## Figures and Tables

**Table 2:** Risk of Bias.

	Selection Bias	Study Design	Confounders	Blinding	Data Collection Method	Withdrawals and Dropouts	Global Rating
Reese et al. (2012) [34]	Weak	Weak	Weak	Weak	Strong	Strong	Weak
Reese et al. (2014) [36]	Weak	Strong	Strong	Weak	Strong	Weak	Weak
DuHamel et al. (2016) [38]	Weak	Strong	Strong	Weak	Strong	Weak	Weak
Brotto et al. (2017) [31]	Weak	Weak	Weak	Weak	Strong	Weak	Weak

## Data Availability

No new data were created or analyzed in this study. Data sharing is not applicable to this article.
